# Editorial: Exploiting cellular immunometabolism as a strategy for innovative cardiovascular therapies

**DOI:** 10.3389/fcvm.2024.1435850

**Published:** 2024-05-31

**Authors:** F. Bonacina, D. Della-Morte

**Affiliations:** ^1^ Department of Excellence of Pharmacological and Biomolecular Sciences “Rodolfo Paoletti”, Università Degli Studi di Milano, Milan, Italy; ^2^ Department of Biomedicine and Prevention, University of Rome Tor Vergata, Rome, Italy; ^3^ Department of Neurology, Miller School of Medicine, University of Miami, Miami, FL, United States

**Keywords:** cardiovascular disease (CVD), atherosclerosis, immune response, immunometabolism, immunomodulation

**Editorial on the Research Topic** Exploiting cellular immunometabolism as a strategy for innovative cardiovascular therapies

In the last few years, immuno-inflammation has emerged as one of the driving risk factors for cardiovascular disease. (CVD) (1). This picture is well illustrated by atherosclerosis, a chronic inflammatory lipid-driven disease of the arteries and a major cause of CVD; in this context, the heterogenous activation of the immune response has indeed been shown to be not just bystander to lipid overload, but instead active player of disease progression (2). This is supported by the observation that CVD patients have systemic alterations in the number, proportion and function of immune cells, showing a pro-inflammatory activation that already occurs at the level of haematopoietic precursors in the bone marrow through mechanisms of functional priming, associated with innate trained immunity, and/or somatic mutations in progenitor cells leading to clonal haematopoiesis of indeterminate potential (CHIP) (3–6). In line with this, neutrophil counts have recently been causally associated to CVD by both observational and genetic approaches (7). This calls for the inclusion of loss of the immuno-inflammatory balance as a risk factor for CVD, and encourages the implementation of therapeutic approaches in the clinic to limit this immuno-inflammatory response (8).

Seminal studies in animal models of atherosclerosis have demonstrated the close relationship between hypercholesterolemia and inflammation in the atherosclerotic plaque. This is also mediated by the excessive release of activated monocytes from the bone marrow and the spleen, which eventually accumulate in the plaque where they differentiate into macrophages (9). The increased proliferation of haematopoietic cells under hypercholesterolemic conditions is linked to changes in sterol metabolism, due to cellular accumulation of cholesterol, and energy metabolism, as a result of a metabolic shift toward glycolysis. The crosstalk between immune cell activation and metabolic adaptations has been further demonstrated in humans by positron emission tomography/computed tomography (PET/CT) imaging using 18F-fluorodeoxyglucose (18F-FDG). In fact, 18F-FDG uptake, which reflects glucose metabolism—particularly increased in metabolically active cells—can non-invasively assess arterial inflammation, which is mainly caused by macrophage uptake in atherosclerotic plaques. Increased 18F-FDG uptake was found in the aorta, bone marrow and spleen of dyslipidaemic patients compared with normocholesterolaemic subjects and was associated with inflammatory biomarkers (10). These observations have also been extended to patients with acute myocardial infarction (11), thus suggesting an increased metabolic activity in these haematopoietic lymphoid districts associated with vascular inflammation, fostering an increased interest in the use of more specific tracers and their combination to better stratify inflammatory risk in CVD patients (12).

This scenario poses the challenge of investigating how the plasticity of cellular metabolism influences the function of cells in the atherosclerotic plaque, leading to the identification of novel molecular pathways that can be targeted to correct the immune-inflammatory response in CVD (13).

From the bench side, cutting-edge research has identified cellular metabolic “checkpoints” whose activity is coupled to functional cellular reprogramming of immune cells. This is the case of the various modulators of cholesterol metabolism, such as the apolipoprotein E, ABCA1 and ABCG1 transporters, the LDL receptor, which have been shown to modulate cellular sterol metabolism in haematopoietic precursors, macrophages, dendritic cells and lymphocytes, in addition to their effect on systemic lipidaemia, thereby controlling cellular functions (14–18). Similarly, modulation of glucose and amino acid metabolism in haematopoietic cells differentially modulates the progression of atherosclerosis [reviewed in detail (19)] In parallel, the identification of autoimmunity against modified lipoproteins (20) (particularly against naïve and oxidised LDL and its protein and lipid components) has set the stage for testing atherosclerosis vaccines in clinical trials with the gaol to stimulate the production of antibodies against LDL, training antigen-specific or vasculotropic immunosuppressive T regulatory cells (21, 22). Indeed, the safety of low-dose IL-2 to promote Treg expansion has been demonstrated in patients with stable and acute CVD (23) and is now being tested for clinical benefit (24). While promising, these therapies rely on the patient's immune system, which may carry genetic or epigenetic “scars” that would potentially limit their clinical efficacy. In this setting, cellular immunotherapies based on ex vivo engineered T cells may overcome this limitation, also thanks to their rapid expansion beyond haematological cancers; indeed, recent experimental data suggest that this approach protects against experimental age-related metabolic dysfunction (25), paving the way to for the use of immune cell therapy in the context of cardiovascular and metabolic diseases.

On the other hand, clinical evidence has shown that lipid-lowering interventions have a cardiovascular benefit that goes beyond improving the plasma lipid profile and may be associated with a reduction in inflammatory burden (26). However, it remains to be proven whether the direct effect can be achieved *in vivo* (after liver metabolism and distribution), as shown in *in vitro* studies. In line with this, the recent evidence for the beneficial effects of SGLT2 inhibitors on cardiac function in patients with or without diabetes (T2M) has been linked to direct anti-inflammatory and immunomodulatory properties, beyond the improved systemic metabolic phenotype (27, 28).

So, what can we learn from these experimental and clinical evidence to improve CVD stratification and treatment? Cutting-edge technologies are improving our understanding of the function, localisation and metabolism of different cell subsets within the atherosclerotic plaque, helping to identify cell-specific immune and/or metabolic dysfunctions that drive cardiovascular inflammation. Therefore, innovative therapeutic approaches may, on the one hand, take advantage of the repositioning of “metabolic drugs” to target molecular “checkpoints” that couple the reprogramming of the cellular energy machinery with its functional modulation, and, on the other hand, adopt a tailored strategy to target specific subset/cells associated with atheroslcerosis-severity, thereby limiting the side effects of off-target modulation of the immune response.
